# Intrinsic anti-inflammatory nanomedicines for enhanced pain management

**DOI:** 10.3389/fbioe.2024.1514245

**Published:** 2024-12-16

**Authors:** Bin Qiao, Jiaqian Yao, Yu’ang Fan, Na Zhang, Miao Feng, Jiaju Zhao, Xinye Song, Yong Luan, Bowen Zhuang, Nan Zhang, Xiaoyan Xie, Ming Xu

**Affiliations:** ^1^ Department of Medical Ultrasonics, The First Affiliated Hospital of Sun Yat-sen University, Guangzhou, China; ^2^ Department of Anesthesiology, The First Affiliated Hospital of Dalian Medical University, Dalian, Liaoning, China

**Keywords:** pain, inflammation, levobupivacaine, nanomedicine, ginsenoside Rg3

## Abstract

**Introduction:**

Effective postoperative pain management remains a significant challenge due to the severe side effects of opioids and the limitations of existing analgesic delivery systems. Inflammation plays a critical role in pain exacerbation, highlighting the need for therapies that combine analgesic effects with intrinsic anti-inflammatory properties.

**Methods:**

Herein, we develop an intrinsic anti-inflammatory nanomedicine designed to enhance pain management by integrating controlled anesthetic release with inherent anti-inflammatory activity. Our nanoplatform utilizes dendritic mesoporous silica nanoparticles (MSNs) loaded with levobupivacaine and coated with Rg3-based liposomes derived from ginsenoside Rg3, termed LMSN-bupi.

**Results:**

The MSNs enable sustained and controlled release of the local anesthetic, while the Rg3-liposome coating provides intrinsic anti-inflammatory effects by inhibiting macrophage activation. In animal models, LMSN-bupi demonstrates significantly prolonged analgesic effects and attenuated inflammatory responses compared to traditional liposome-decorated nanoparticles (TMSN-bupi) (n = 5).

**Discussion:**

These findings underscore the potential of intrinsic anti-inflammatory nanomedicines in enhancing pain management, offering a promising strategy to overcome the limitations of current therapies and improve patient outcomes in postoperative care.

## 1 Introduction

Effective postoperative pain management continues to pose a formidable global health challenge, stemming from its widespread occurrence and the severe consequences tied to current analgesic treatments (Fitzcharles et al., 2021). Approximately 2%–10% of these individuals will experience severe or chronic pain (Neuman et al., 2021). Consequently, persistent postoperative pain represents a major, yet largely overlooked, clinical challenge (Kehlet et al., 2006). Addressing and preventing the onset of long-term pain following surgery is a crucial aspect of patient-centered healthcare. Opioid receptor agonists like morphine and its derivatives are currently the mainstay for postoperative pain relief, providing potent analgesia by activating the μ-opioid receptor (Colvin et al., 2019; Stein, 2016). However, the prolonged administration of opioids sensitizes the central nervous system (CNS), resulting in debilitating side effects that encompass constipation, sedation, tolerance development, physical dependency, and respiratory depression (Pirie et al., 2022). The escalating opioid epidemic—with 15.5 million opioid-dependent people globally in 2010 and over 80,000 deaths attributed to opioid-related overdose in the United States in 2021 alone—underscores the urgent need for alternative pain management strategies that offer effective analgesia while minimizing adverse outcomes (Hazam et al., 2024; Degenhardt et al., 2014).

Recent research efforts have focused on extending drug duration or enhancing therapeutic efficacy through external stimuli to improve postoperative pain management (Qiao et al., 2023; Bhansali et al., 2021; Ouyang et al., 2020; Zhang et al., 2020). Advanced drug delivery systems hold potential for reducing opioid consumption and enabling tunable pain control (Feng et al., 2024; Qiao et al., 2022; Babaie et al., 2022). However, clinical translation of these approaches faces significant challenges. External stimulation techniques, such as ultrasound or photothermal therapy, are often impractical for consistent administration in clinical settings (Rwei et al., 2017; Song et al., 2022; Liu et al., 2020). Moreover, anesthetic delivery systems designed to prolong drug action can inadvertently trigger immune responses, leading to inflammation that exacerbates pain (Le Franc et al., 2024; Ji et al., 2014; Sun et al., 2022; Xie et al., 2023). This inflammatory response undermines the therapeutic potential of nanomedicine-based delivery systems and limits their clinical utility (Luo et al., 2022).

Anti-inflammatory treatments offer a promising solution by mitigating pain driven by inflammation and reducing the likelihood of central sensitization (Conaghan et al., 2019; Zhang et al., 2022; Guo et al., 2023). Combining anti-inflammatory agents with local anesthetics not only prolongs analgesia but also reduces the required dosage of anesthetics, thereby minimizing side effects (Nestor et al., 2022; Carley et al., 2021; Lim et al., 2024). This synergistic approach aligns with the need for multimodal pain management strategies, leveraging the complementary actions of different agents to optimize clinical outcomes. Integrating anti-inflammatory therapies with advanced drug delivery systems thus represents a promising avenue for achieving more effective and patient-friendly postoperative pain management.

In our recent studies, we demonstrated the potential of dendritic mesoporous silica nanoparticle (MSN)-based drug delivery systems to develop safe and effective analgesic nanomedicines by encapsulating levobupivacaine, a widely used clinical anesthetic (Qiao et al., 2023). However, these efforts have primarily focused on local anesthetics for pain management, overlooking the inherent immune activation triggered by nanomaterials like MSNs, for which effective mitigation strategies remain limited (Sun et al., 2021). Additionally, the analgesic duration provided by silica-based nanomaterials remains suboptimal, necessitating further improvements. To address these challenges, surface modification of nanocarriers with liposomes has emerged as a promising strategy to enhance therapeutic performance (Guo et al., 2022; Jiang et al., 2022). Specifically, Rg3-based liposomes, derived from ginsenoside Rg3, have demonstrated the ability to prevent macrophage activation and reduce nanoparticle uptake *in vivo*, thereby mitigating inflammatory responses (Zhu et al., 2023; Ren et al., 2021; Li et al., 2023; Xia et al., 2022). We hypothesize that decorating silica nanomedicines with Rg3-based liposomes could effectively suppress the inflammatory effects induced by the nanocarrier and extend the duration of analgesia.

Here, we report the development of a nanomedicine for postoperative pain management that combines local anesthetic delivery with intrinsic anti-inflammatory effects. We employed Rg3-based liposomes to enhance anti-inflammatory activity at the targeted site. Compared to traditional liposome-coated nanoparticles, Rg3-based liposomes show potential for greater inflammation reduction and prolonged pain relief. Using mesoporous silica nanoparticles (MSNs) as the platform, we developed Rg3-liposome-modified MSNs loaded with levobupivacaine (LMSN-bupi), enabling sustained release and enhanced analgesia. LMSN-bupi exhibited superior pain management and anti-inflammatory effects compared to traditional liposome-decorated MSN-bupi (TMSN-bupi). These findings demonstrate the potential of intrinsic anti-inflammatory nanomedicines to improve pain management and patient outcomes.

## 2 Materials and methods

### 2.1 Materials

Rg3, levobupivacaine hydrochloride, and ethanol were obtained from Aladdin. PL-100M and cholesterol were purchased from AVT (Shanghai) Pharmaceutical Tech Co. Ltd. Triethanolamine, bis [3-(triethoxysilyl)propyl] tetrasulfide, cetyltrimethylammonium chloride, as well as tetraethyl orthosilicate, were all acquired from Sigma-Aldrich (United States). Endothelial cell medium was supplied by Sciencell Corporation. The standard CCK-8 assay kit was acquired from Dojindo Molecular Biotechnologies Co., Ltd (Japan). Dialysis bags featuring a 3,500 Da molecular weight cutoff were supplied by Yuanye Biotechnology Co., Ltd.

### 2.2 Characterizations

Transmission electron microscopy images of MSN, MSN-bupi, and LMSN-bupi were acquired using a Hitachi H-7650 instrument, which was operated at 50 kV. Dynamic Light Scattering (DLS) measurements were performed in distilled water utilizing the Litesizer500 instrument (Anton Paar) to ascertain precise particle size distributions. Nitrogen adsorption-desorption isotherms were acquired utilizing a Micromeritics ASAP 2460 analyzer to assess the pore size distribution and determine the Brunauer-Emmett-Teller (BET) specific surface area subsequent to degassing the samples at 120°C for a duration of 6 h. The absorption spectra spanning the ultraviolet-visible-near infrared (UV-vis-NIR) range were precisely recorded utilizing a Shimadzu UV-1900i spectrophotometer.

### 2.3 Synthesis of dendritic mesoporous silica nanoparticles (MSN)

MSN were synthesized adhering to a previously documented protocol (Wu et al., 2015). Specifically, 2.0 g of CTAC and a suitable quantity of triethanolamine (TEA) were dissolved in 20 mL of distilled water while stirring at 95°C. After a period of 20 min, 1.0 g of TEOS and 1.3 g of BTES were gradually introduced to the mixture, which was then stirred for an additional 4 h. The subsequent product underwent rigorous washing with ethanol and water to eliminate residual reactants. To remove CTAC, the product was refluxed in a 10% (v/v) hydrochloric acid solution in ethanol at 78°C for a duration of 12 h.

### 2.4 Loading levobupivacaine into MSN

A quantity of 10 mg of MSN was dispersed in 5 mL of distilled water. Following this, 10 mg of levobupivacaine was incorporated into the suspension, and the mixture was stirred at room temperature overnight. The suspension was centrifuged at 12,000 rpm for 10 min, and the resultant precipitate was collected, designated as MSN-bupi.

### 2.5 Fabrication of Rg3-integrated and conventional liposomes

The phospholipid PL-100M and Rg3 (alternatively, cholesterol for conventional liposomes) were intimately blended at a precise weight ratio of 10:3 and subsequently dissolved in chloroform. The solvent was then subjected to vacuum evaporation at a controlled temperature of 48°C, resulting in the formation of a delicate lipid film. This film was subsequently hydrated with PBS solution, ultimately yielding the desired liposomes.

### 2.6 Preparation of LMSN-bupi

The encapsulation of MSN-bupi nanoparticles within liposomes was achieved through an extrusion method employing a mini-extruder sourced from Avanti Polar Lipids, United States (Xia et al., 2022) Initially, 0.9 mg of pre-fabricated MSN-bupi nanoparticles underwent extensive rinsing with distilled water and were then dispersed in 3 mL of ultrapure water. Concurrently, 0.9 mg of liposome membranes were solubilized in 400 μL of ultrapure water and introduced into the MSN-bupi dispersion. This mixture was then subjected to 11 cycles of extrusion through an 800 nm porous filter membrane using the Avanti mini-extruder. Following this, the resultant dispersion was centrifuged at 12,000 rpm for 10 min at 4°C, and subsequently rinsed three more times with chilled distilled water. The supernatant was then collected for UV-vis spectroscopic analysis to determine the concentration of levobupivacaine. The loading efficiency of levobupivacaine was calculated using the formula: Loading Efficiency = (1 − [levobupivacaine in supernatant]/[total levobupivacaine]) × 100%.

### 2.7 *In Vitro* release studies of levobupivacaine

To assess the release characteristics of levobupivacaine from MSN-bupi, LMSN-bupi, and TMSN-bupi nanoparticles, a concentration of 5 mg/mL was encapsulated within dialysis bags featuring a molecular weight cutoff of 3.5 kDa. These bags were then submerged in 20 mL of distilled water, with the entire system maintained at a stable temperature and agitated at 160 rpm utilizing a JINGHONG shaker. At predetermined time points, 5 mL aliquots of the release medium were extracted for levobupivacaine quantification, and an equivalent volume of fresh distilled water was replenished to sustain sink conditions. The cumulative release was subsequently calculated using standard calibration curves derived from UV-vis absorbance measurements.

### 2.8 Cytotoxicity evaluation

Using the standardized CCK-8 assay, we evaluated the cytotoxicity of LMSN-bupi and TMSN-bupi on Dorsal Root Ganglion (DRG) cells sourced from BIOSPECIES. Initially, DRG cells were seeded into 96-well plates and cultured overnight. Following the removal of the culture medium, the cells were exposed to varying concentrations of LMSN-bupi and TMSN-bupi in ECM medium and incubated for durations of 12 or 24 h. At the predetermined time points, the medium was substituted with 100 μL of a freshly prepared 10% CCK-8 solution diluted in fresh medium, and the cells were further incubated for an additional 1–2 h. Cell viability was subsequently quantified by recording the absorbance at 450 nm utilizing a microplate reader (Varioskan LUX, Thermo Scientific Inc., United States).

### 2.9 Animals

Male Balb/c mice, aged between 4 and 6 weeks, were procured from Liaoning Changsheng Biotechnology Corporation. The Animal Care Committee of Dalian Medical University granted approval for all animal experiments, which were conducted strictly adhering to the institutional guidelines. Prior to undergoing behavioral testing, the mice were housed in a temperature-regulated environment and allowed to acclimate for a period of 2 weeks.

### 2.10 Establishment of mouse incision pain model

A previously described model for assessing pain in mice through incision was established (Pogatzki and Raja, 2003). Mice were anesthetized with 2% isoflurane. Prior to surgery, the surgical area was thoroughly disinfected using a 10% povidone-iodine solution. A 2 mm longitudinal incision was precisely made, initiating from the proximal edge of the heel. The underlying muscle tissue was gently lifted and incised along its entire length. Subsequently, the skin incision was meticulously closed using 4–0 sutures, and an antibiotic ointment was applied to the wound site to prevent potential infection.

### 2.11 *In Vivo* drug administration

Mice were randomly assigned to groups and received a single local injection (100 μL) around the sciatic nerve (n = 5 per group). The treatments included MSN-bupi suspension, LMSN-bupi suspension, TMSN-bupi suspension, levobupivacaine solution (containing 100 μg of levobupivacaine), or PBS as a control.

### 2.12 Measurement of mechanical allodynia

Mechanical allodynia was assessed utilizing the von Frey filament test (KW-CT, Kaerwen Inc., China) to evaluate the response to non-noxious mechanical stimuli (Mohd Isa et al., 2018). Mice were positioned on a wire mesh floor within transparent enclosures and given at least 30 min to acclimatize. A probe was gently applied to the plantar surface of the hind paw, incrementally increasing the force applied until a withdrawal response was elicited. The force level at which the withdrawal occurred was documented as the paw withdrawal threshold (PWT). Each mouse underwent a series of 5 trials, and the mean PWT was subsequently calculated for statistical analysis.

### 2.13 Measurement of thermal hyperalgesia

Thermal hyperalgesia was evaluated utilizing a hot plate test apparatus (KW-LB, Kaerwen Inc.) maintained at a temperature of 55°C (Wang et al., 2022). Mice were individually placed on the hot plate, and the paw withdrawal latency (PWL)-the time taken for the injured hind paw to be lifted-was automatically recorded. Three measurements were taken for each mouse with intervals of 5–10 min to prevent tissue damage, and a cutoff time of 20 s was established. The average PWL was used for statistical analysis.

### 2.14 Cytokines and histological staining analysis

Mice underwent euthanasia at 3 and 7 days post-treatment with LMSN-bupi and TMSN-bupi. Subsequently, the sciatic nerve and adjacent tissues were excised and immersed in 4% paraformaldehyde overnight for fixation. These tissues underwent hematoxylin and eosin (H&E) staining, while additional sciatic nerve sections were specifically stained with toluidine blue. An automated digital slide scanner from KFBIO Inc. facilitated the analysis of the stained sections. Furthermore, tissue samples were procured for cytokine assays.

### 2.15 Statistical analysis

Statistical analyses were performed using GraphPad Prism 9 software. Data are presented as mean ± standard deviation. Comparisons between two groups were made using Student’s t-test, while one-way ANOVA was used for multiple group comparisons. A *p*-value of less than 0.05 was considered statistically significant.

## 3 Results

### 3.1 Preparation and characterization of liposome-camouflaged MSN-bupi

**FIGURE 1:**
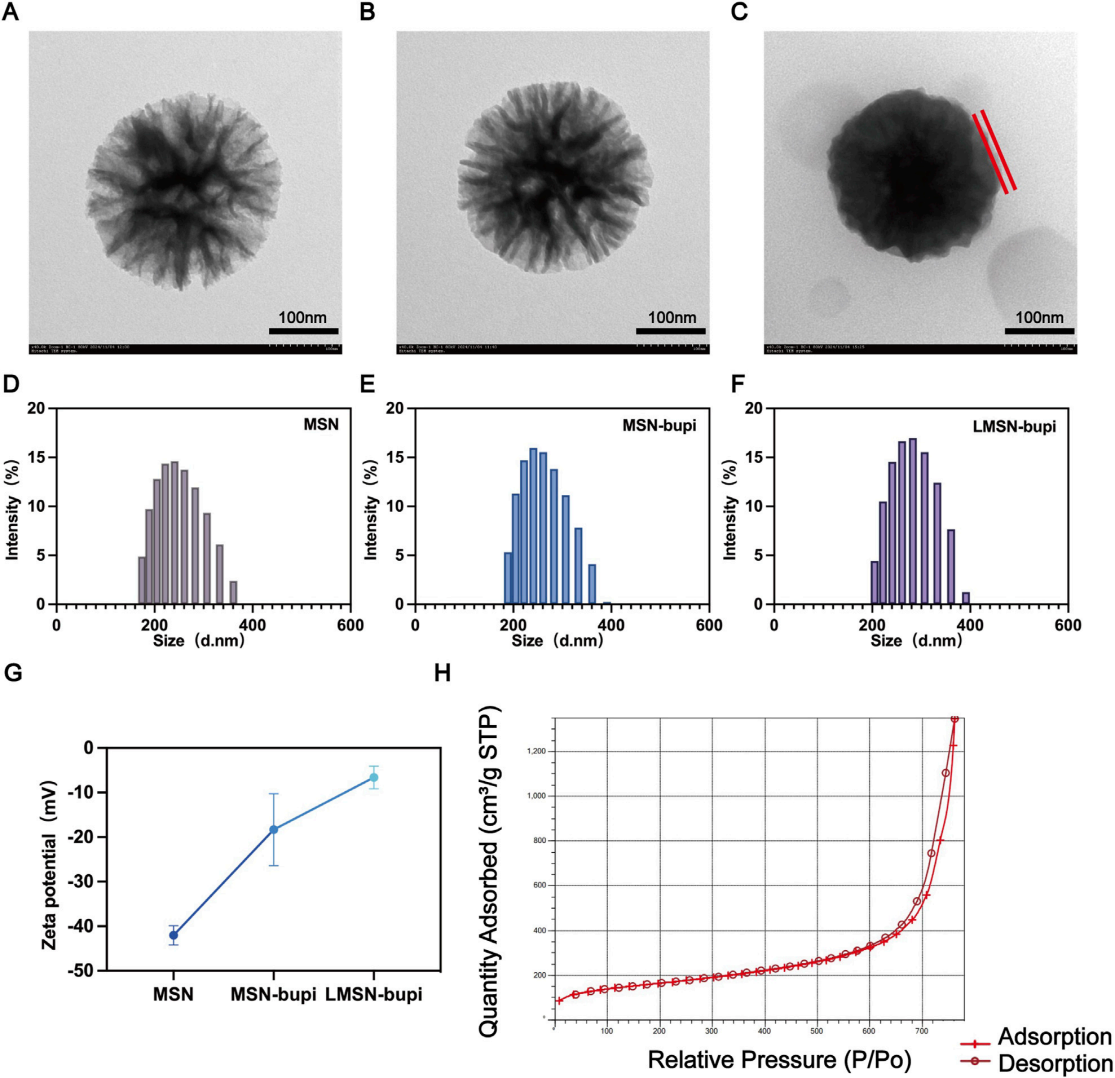
Representative TEM images of **(A)** MSN, **(B)** MSN-bupi, and **(C)** LMSN-bupi. The scale bars are 200 nm. The liposomes were labeled. Size distribution of **(D)** MSN, **(E)** MSN-bupi, and **(F)** LMSN-bupi. **(G)** Zeta potentials of MSN, MSN-bupi, and LMSN-bupi. **(H)** BET result of MSN.

Rg3-based liposomes and conventional liposomes were prepared using the thin-film hydration method as previously described. Dendritic mesoporous silica nanoparticles (MSN) were synthesized following established protocols. Transmission electron microscopy (TEM) images confirmed that the synthesized MSN exhibited a spherical morphology with large pores (Figure 1A). To enhance pain management capabilities, levobupivacaine hydrochloride was loaded into MSN to form MSN-bupi nanoparticles. TEM images showed increased opacity within the particles after levobupivacaine loading, indicating that the inner pores were filled (Figure 1B). To fabricate liposome-camouflaged MSN-bupi (LMSN-bupi) incorporating Rg3, a blend of Rg3-based liposomes and MSN-bupi was extruded through a mini-extruder for a total of 11 passes. TEM images of the resulting LMSN-bupi nanoparticles displayed spherical core–shell structures, confirming successful coating with Rg3-based liposomes (Figure 1C) (Qiao et al., 2020) Dynamic light scattering (DLS) analysis revealed an increase in hydrodynamic diameter for LMSN-bupi (280 nm, PDI 0.256) compared to uncoated MSN (247 nm, PDI 0.318) and MSN-bupi (259 nm, PDI 0.197), indicating successful liposome coating (Figures 1D–F). The zeta potential shifted from −42.03 ± 1.7 5 mV for MSN to approximately −18.33 ± 6.59 mV after loading with levobupivacaine to form MSN-bupi, and further to −6.6 ± 2.1 mV upon coating with Rg3-based liposomes to produce LMSN-bupi (Figure 1G). Nitrogen adsorption-desorption isotherms were measured to confirm the mesoporous structure of MSN (Figures 1H, I). The hysteresis loops observed are characteristic of mesoporous materials. The Brunauer-Emmett-Teller (BET) surface area (553 mm^2^/g) and average pore diameter (18.3 nm) indicated a high porosity of the MSN. Based on the UV-vis-NIR absorption spectra analysis, the encapsulation efficiency of levobupivacaine within the MSN-bupi matrix was precisely determined to be 86.4%.

### 3.2 *In Vitro* levobupivacaine release profile

**FIGURE 2:**
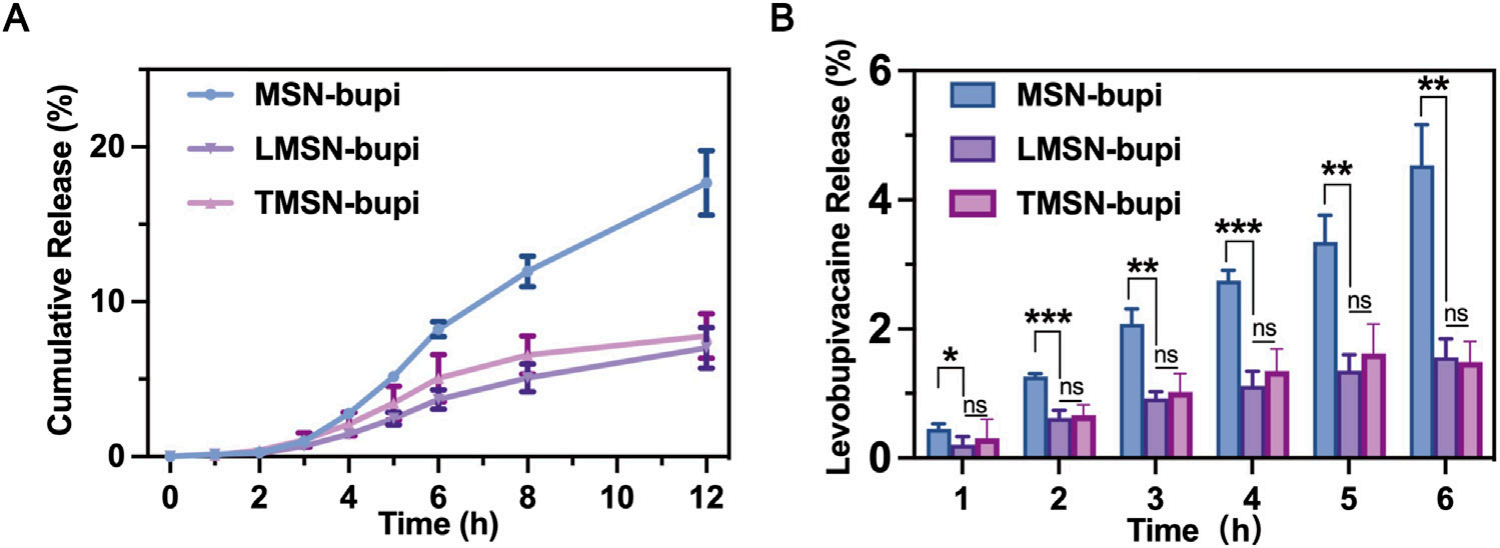
**(A)** Cumulative release of MSN-bupi, LMSN-bupi and TMSN-bupi. **(B)** Cumulative release quantification of MSN-bupi, LMSN-bupi, and TMSN-bupi. The data was shown as mean ± SD, n = 5 per group, ∗*p* < 0.05, ∗∗*p* < 0.01, ∗∗∗*p* < 0.001.

The liposome camouflaging of the nanoparticles may have contributed to plugging the pores of the MSN, thereby enhancing the sustained release of levobupivacaine. To evaluate this effect, levobupivacaine release profiles from different nanoparticles were observed at predetermined time points. Notably, the differences in levobupivacaine release between LMSN-bupi and MSN-bupi highlight the impact of liposome decoration (Figure 2A). Under identical conditions, LMSN-bupi exhibited a slower release of levobupivacaine compared to MSN-bupi. Importantly, no significant variation was observed between LMSN-bupi and TMSN-bupi, indicating that the drug release process was primarily governed by the liposome coating rather than the specific liposome composition. At specific time points, the cumulative release of levobupivacaine from the MSN-bupi group was significantly higher than that from the LMSN-bupi and TMSN-bupi groups (Figure 2B). This sustained release characteristic of LMSN-bupi nanoparticles is advantageous for prolonged pain management.

### 3.3 *In Vitro* biocompatibility of LMSN-bupi and TMSN-bupi

**FIGURE 3:**
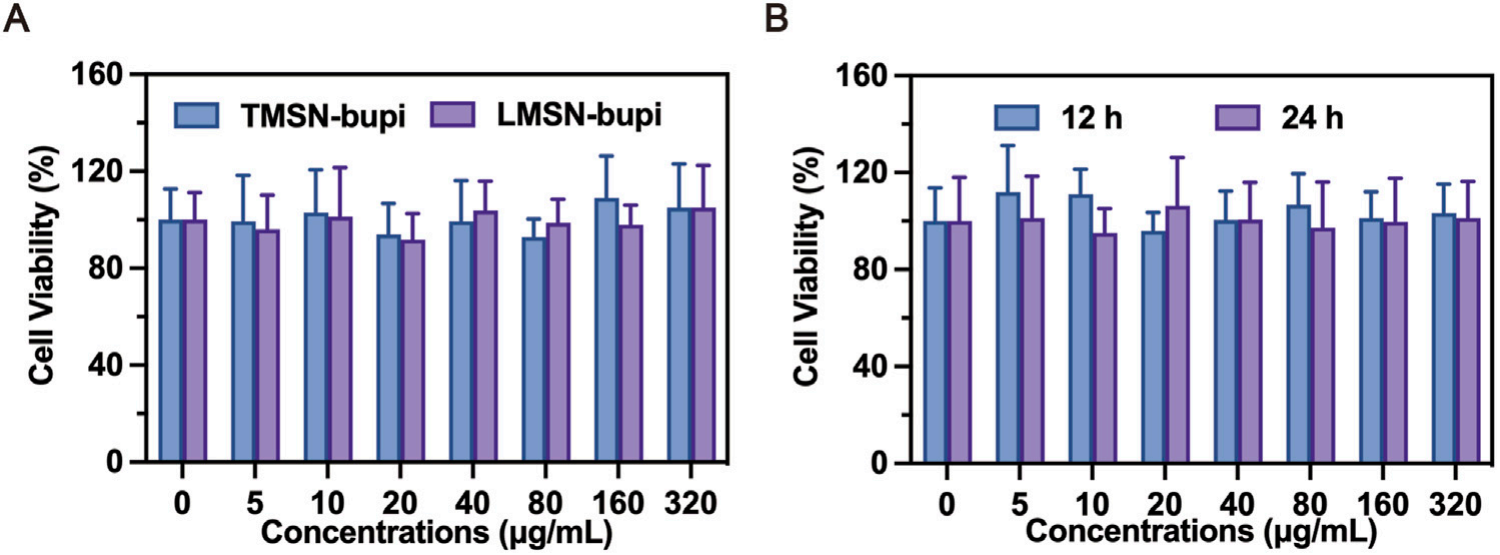
**(A)** Cell viability assay of LMSN-bupi and TMSN-bupi incubated with DRG cells. **(B)** Cell viability assay of LMSN-bupi incubated with DRG cells for 12 and 24 h.

The cytotoxicity of LMSN-bupi and TMSN-bupi toward dorsal root ganglion (DRG) cells was assessed using the standard CCK-8 assay (Liang et al., 2022). DRG cells were chosen due to their representative nature of normal cells that play a crucial role in the transmission of pain. After a 24-hour co-incubation period with LMSN-bupi and TMSN-bupi, the results of the cell viability assays revealed no statistically significant difference between the treated and control groups, suggesting that these nanoparticles had no detrimental effect on the growth of DRG cells (Figure 3A). Furthermore, DRG cells co-incubated with LMSN-bupi for 12 h and 24 h exhibited unchanged viability (Figure 3B), corroborating the high biocompatibility of LMSN-bupi for pain management applications.

### 3.4 *In Vivo* pain management

**FIGURE 4:**
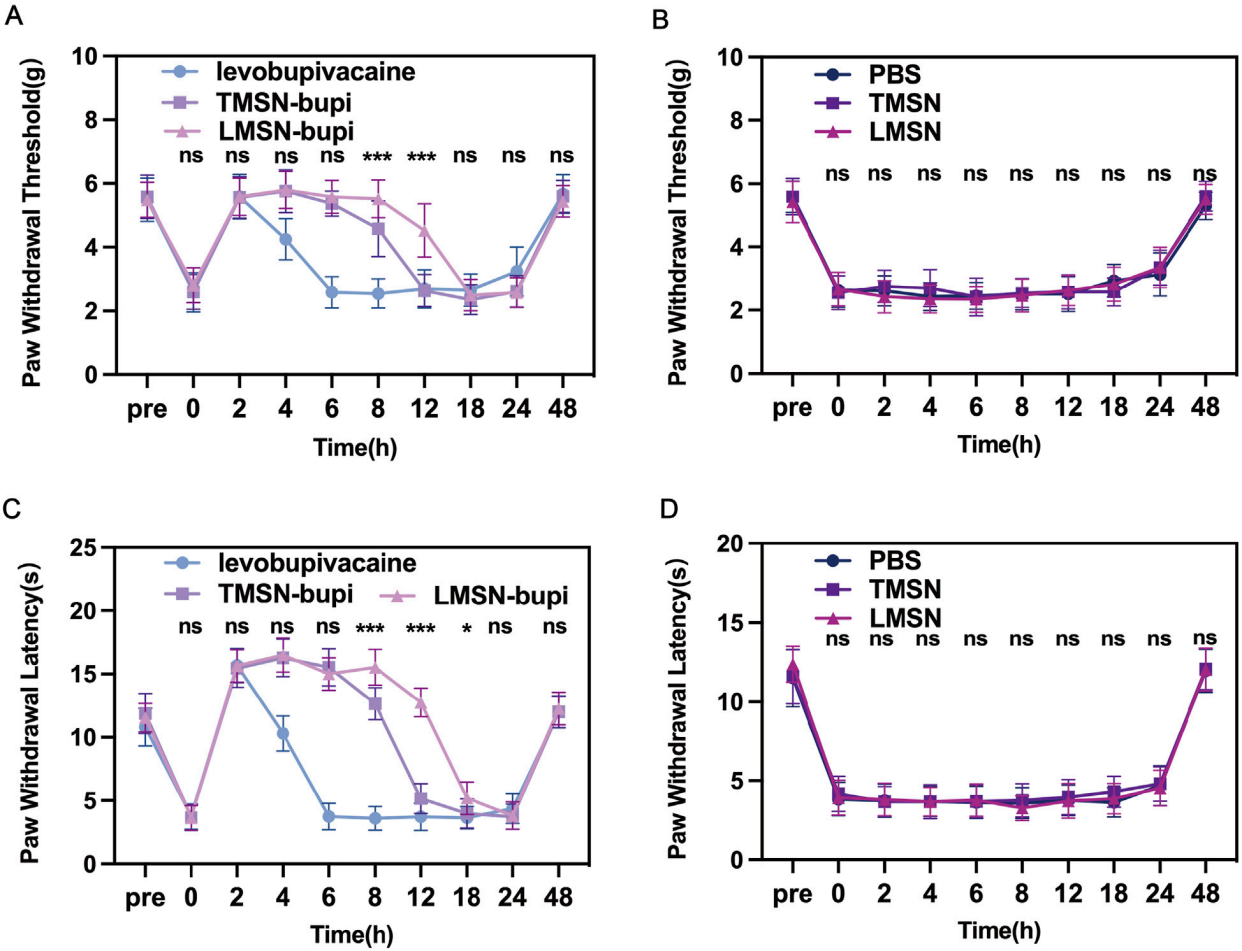
**(A)** Paw withdrawal threshold of mice treated by Levobupivacaine, TMSN-bupi, and LMSN-bupi. **(B)** Paw withdrawal threshold of mice treated by PBS, TMSN, and LMSN. **(C)** Thermal latency of mice treated by Levobupivacaine, TMSN-bupi, and LMSN-bupi. **(D)** Thermal latency of mice treated by PBS, TMSN, and LMSN. The data was shown as mean ± SD, n = 5 per group, between the TMSN-bupi group and the LMSN-bupi group, ∗*p* < 0.05, ∗∗*p* < 0.01, ∗∗∗*p* < 0.001.

The efficacy of LMSN-bupi nanoparticles for pain management was evaluated *in vivo* using a mouse incision pain model. Three hours following surgery, a significant decrease in mechanical threshold and thermal latency was observed, confirming the successful induction of pain. Subsequently, the mice underwent perineural injection of levobupivacaine, TMSN-bupi, or LMSN-bupi in the vicinity of the sciatic nerve. To assess mechanical hyperalgesia, behavioral tests were conducted utilizing mechanical stimulation devices that precisely measured the mechanical threshold (Figures 4A, B). Following injection, thermal and mechanical pain responses were meticulously monitored at 2-hour intervals. Notably, the injection of 100 μL of PBS (serving as the control) did not elicit any changes in the mechanical threshold (Figure 4A). Conversely, the administration of 100 μL of levobupivacaine effectively produced a pain-blocking effect that persisted for approximately 6 h. In contrast, administration of 100 μL of TMSN-bupi extended the duration of analgesia to about 12 h, highlighting the potential of nanotechnology to prolong the effect of local anesthetics. Notably, administration of 100 μL of LMSN-bupi further extended the analgesic duration up to 18 h compared to TMSN-bupi, which can be attributed to the intrinsic anti-inflammatory properties of Rg3. Mice receiving PBS, TMSN, or LMSN without levobupivacaine showed no analgesic effect, indicating that MSN alone did not contribute to pain management (Figure 4B).

Paw Withdrawal Latency (PWL) under thermal stimulation was evaluated to assess thermal hyperalgesia. Injection of PBS did not alter the thermal response (Figure 4C), whereas the administration of levobupivacaine induced a 6-hour pain blockade. Likewise, mice subjected to sciatic nerve injection with TMSN-bupi displayed a prolonged PWL on a 55°C hot plate for approximately 12 h, in comparison to levobupivacaine administration alone, indicative of enhanced pain management. Notably, LMSN-bupi exhibited a markedly longer duration of analgesia than TMSN-bupi, which can be attributed to the Rg3-based liposome modification that prolongs *in vivo* retention time. In contrast, negligible analgesic effects were observed in the PBS, TMSN, and LMSN groups devoid of levobupivacaine, highlighting that the nanoparticles alone did not contribute to pain alleviation (Figure 4D). By 48 h, both thermal and mechanical thresholds in all groups had reverted to baseline levels, verifying the temporary nature of the analgesic effects and validating the incision pain model.

### 3.5 Histocompatibility and neurotoxicity assessment

**FIGURE 5:**
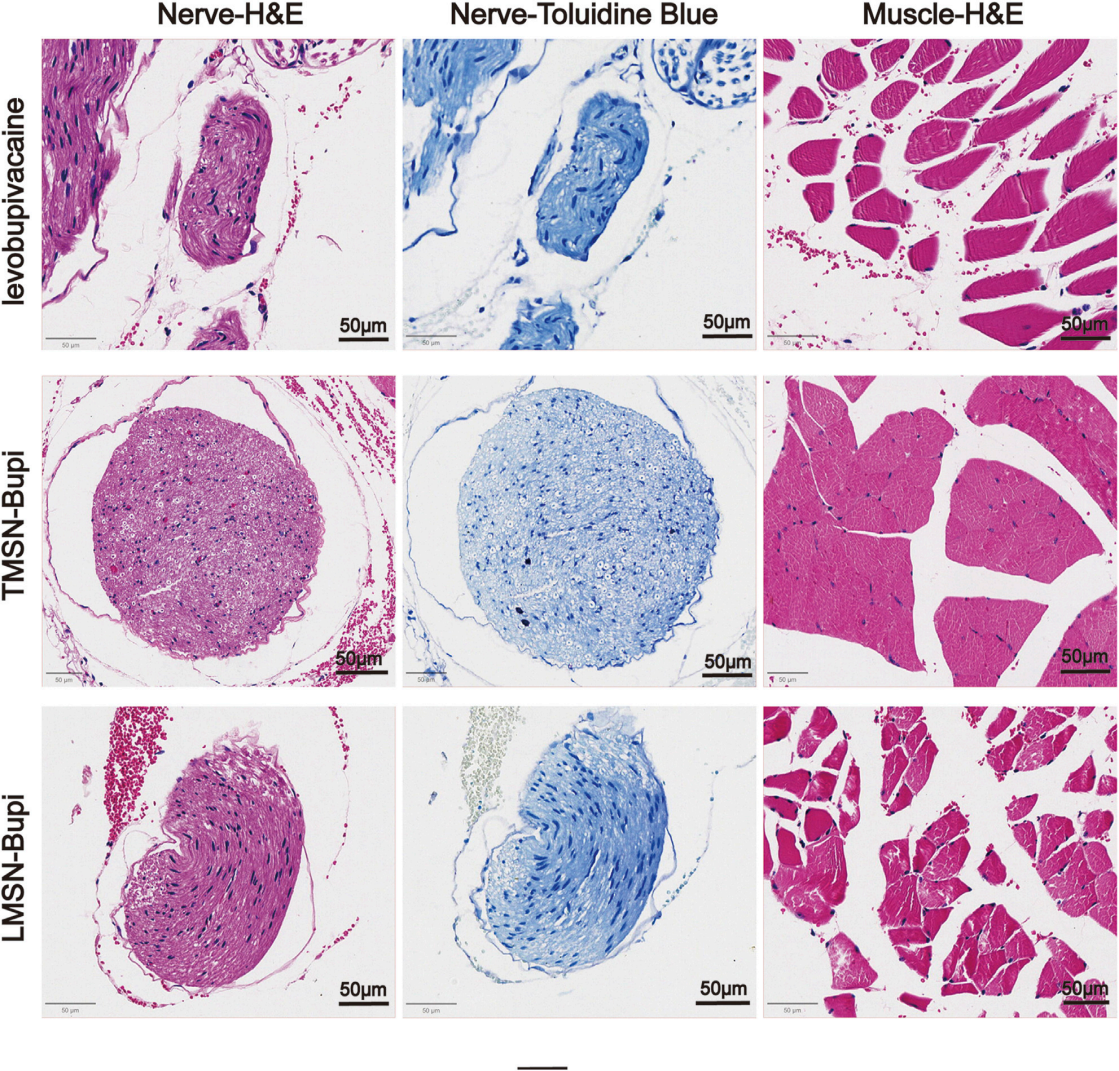
Representative H&E and toluidine blue-stained images of sciatic nerve, skin, and muscle.

To evaluate the biosafety of LMSN-bupi in managing pain, mice that received LMSN-bupi injections were euthanized 7°days post-administration. The sciatic nerve, along with adjacent muscle and skin tissues, were collected and underwent hematoxylin and eosin (H&E) staining as well as toluidine blue staining (Bai et al., 2024). Histological analysis indicated an absence of swelling, discoloration, or tissue damage (Figure 5). Recognizing the potential limitations of H&E staining in detecting nerve injury, toluidine blue staining was additionally employed on sciatic nerve sections. Toluidine blue specifically stains Nissl bodies, serving as a marker of neuronal integrity. After 7 days, no notable peripheral nerve damage was detected. These findings underscore the exceptional biocompatibility of LMSN-bupi nanoparticles in the context of pain therapy.

### 3.6 Modulation of inflammatory cytokines

**FIGURE 6:**
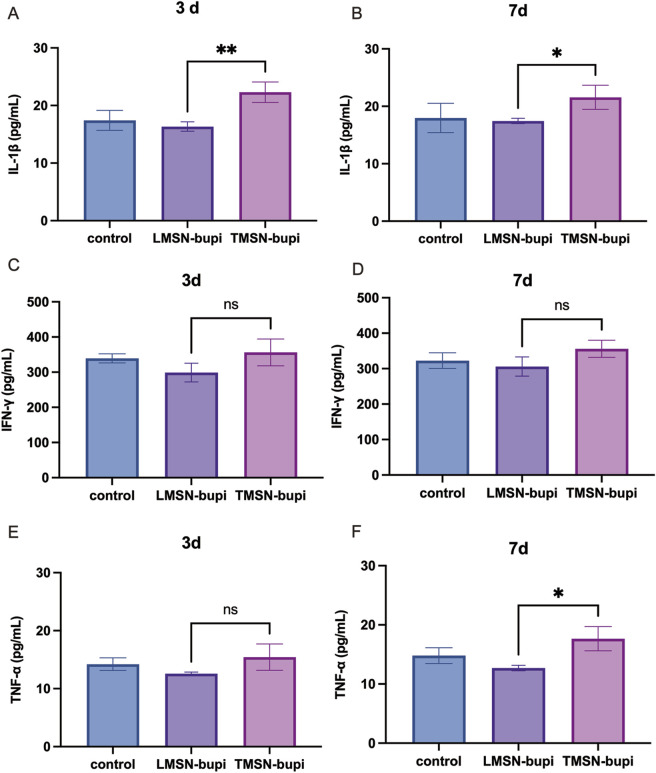
**(A, B)** Cytokines level of IL-1β of sciatic nerve, skin, and muscle at **(A)** 3 days and **(B)** 7 days. **(C, D)** Cytokines level of IFN-γ of sciatic nerve, skin, and muscle at **(C)** 3 days and **(D)** 7 days. **(E, F)** Cytokines level of TNF-α of sciatic nerve, skin, and muscle at **(E)** 3 days and **(F)** 7 days. The data was shown as mean ± SD, n = 3 per group, *p < 0.05, **p < 0.01.

To examine the impact of LMSN-bupi on inflammatory cytokines during pain management, mice injected with varying nanoparticles were euthanized at 3 and 7°days post-injection. The sciatic nerve, along with adjacent muscle and skin tissues, were harvested for a detailed cytokine analysis using ELISA. When compared to the control group, mice administered with TMSN-bupi displayed heightened concentrations of pro-inflammatory cytokines, including IL-1β, IFN-γ, and TNF-α, indicative of a potential inflammatory response induced by TMSN-bupi (Figures 6A–F) (Wu et al., 2023; Jiang et al., 2024) In contrast, mice treated with LMSN-bupi showed reduced cytokine levels at both 3 and 7°days post-injection, which can be attributed to the anti-inflammatory effects of Rg3. These findings demonstrate that LMSN-bupi nanoparticles not only provide effective pain relief but also modulate inflammatory responses, enhancing the overall efficacy of pain management.

## 4 Conclusion

In summary, we have successfully developed an intrinsic anti-inflammatory nanomedicine designed to enhance postoperative pain management. Our LMSN-bupi nanoplatform, comprising dendritic mesoporous silica nanoparticles loaded with levobupivacaine and coated with Rg3-based liposomes, effectively integrates sustained anesthetic release with anti-inflammatory properties. The Rg3-based liposomes, derived from ginsenoside Rg3, not only attenuate immune activation but also prolong the duration of analgesia. Compared to traditional liposome-decorated nanoparticles, LMSN-bupi demonstrates superior analgesic efficacy and reduced inflammatory response *in vivo*. These findings underscore the potential of combining nanotechnology with intrinsic anti-inflammatory mechanisms to overcome the limitations of current analgesic systems. These findings underscore the potential of intrinsic anti-inflammatory nanomedicines in enhancing pain management, offering a promising strategy to overcome the limitations of current therapies and improve patient outcomes in postoperative care. Further exploration of this platform may also open avenues for multimodal pain management strategies, addressing critical challenges in clinical pain treatment.

## Data Availability

The original contributions presented in the study are included in the article/supplementary material, further inquiries can be directed to the corresponding authors.
